# Expression of EBAG9/RCAS1 is associated with advanced disease in human epithelial ovarian cancer

**DOI:** 10.1038/sj.bjc.6601832

**Published:** 2004-05-26

**Authors:** J-i Akahira, M Aoki, T Suzuki, T Moriya, H Niikura, K Ito, S Inoue, K Okamura, H Sasano, N Yaegashi

**Affiliations:** 1 Department of Obstetrics and Gynecology, Tohoku University Graduate School of Medicine, Sendai, Japan; 2 Department of Pathology, Tohoku University Graduate School of Medicine, Sendai, Japan; 3 Department of Biochemistry, Saitama Medical School, Saitama, Japan

**Keywords:** EBAG9, ovarian cancer, immunohistochemistry, oestrogen receptor

## Abstract

Oestrogen receptor-binding fragment associated gene 9, EBAG9, is an oestrogen-responsive gene that was identified in MCF-7 human breast carcinoma cell line. It is identical to RCAS 1, a cancer cell surface antigen possibly involved in immune escape. In the present study, we examined the expression of EBAG9/RCAS1 in human epithelial ovarian cancer using immunohistochemistry, immunoblotting and reverse transcription–polymerase chain reaction (RT–PCR). A total of 90 epithelial ovarian cancer cases were examined immunohistochemically by means of the antibodies for EBAG9 and ER*α*. The correlation between EBAG9 immunoreactivity and clinicopathological parameters was examined. mRNA expression of EBAG9 and ER*α* were evaluated by RT–PCR in 22 cases. The expression for EBAG9 and ER*α* was examined by immunoblotting in 12 ovarian cancer cell lines. EBAG9 immunoreactivity was detected in the surface and cytoplasm of carcinoma cells in 46 out of 90 cases (51.1%). EBAG9 expression was significantly higher in serous histology (*P*=0.0402) and advanced disease (*P*=0.0206). No significant relationship was detected between EBAG9 immunoreactivity and overall survival (*P*=0.689). There was a highly significant correlation between EBAG9 and ER immunoreactivity (*P*<0.0001). The EBAG9 mRNA was detected in 20 out of 22 cases. In all of the cases that were positive for ER*α* mRNA, they were also positive for EBAG9 mRNA. Immunoreactive band corresponding to EBAG9 was detected in 11 out of 12 of ovarian cancer cell lines, and was consistent with ER*α* expression. In conclusion, the wide distribution of EBAG9 and its relation to advanced disease suggest that this protein may play important roles in epithelial ovarian cancer.

Epithelial ovarian cancer is the leading cause of death from gynaecological malignancies in the great majority of developed countries (Akahira J *et al*, 2001; Akahira JI *et al*, 2001,). Sex steroid hormones have been implicated in the aetiology and/or progression of some epithelial ovarian cancers. Both oestrogen (ER) and progesterone receptors (PR) have been reported in human epithelial ovarian cancer (Rao and Slotman, 1991; Akahira *et al*, 2002). In endometrial and breast carcinomas, steroid hormone receptor status correlates well with response to hormonal manipulation and prognosis (McGuire, 1978; Benraad *et al*, 1980; Ehrlich *et al*, 1981; Kaupilla, 1984). However, in epithelial ovarian carcinoma, the prognostic significance of tumour ER status among patients still remains controversial (Bizzi *et al*, 1988; Masood *et al*, 1989; Sevelda *et al*, 1990; Rao and Slotman, 1991; Hempling *et al*, 1998).

Recently, oestrogen receptor-binding fragment associated gene 9 (EBAG9) has been identified as an oestrogen-responsive gene from a cDNA library of MCF-7 human breast cancer cell line (Watanabe *et al*, 1998). Oestrogen receptor-binding fragment associated gene 9 is identical to the receptor-binding cancer antigen expressed in SiSo cells (RCAS1) (Nakashima *et al*, 1999). The EBAG9/RCAS1 is a membrane molecule that acts as a ligand for a putative receptor present in cells (Nakashima *et al*, 1999). *In vitro* studies have also demonstrated that EBAG9/RCAS1 inhibits growth of activated CD3^+^ T lymphocytes, suggesting a possible involvement in the immune escape of neoplastic cells (Nakashima *et al*, 1999). Endocrine-immune interactions are considered to play an important role in the development and/or progression of various hormone-dependent neoplasms, but the details of these interactions remain unclear.

Oestrogen receptor-binding fragment associated gene 9 is demonstrated to be widely distributed in human breast carcinoma, and may play an important role in the development of this oestrogen-dependent cancer (Suzuki *et al*, 2001). Others reported that RCAS1 is associated with poor prognosis and/or advanced stage in various human cancers (Sonoda *et al*, 1996; Kaku *et al*, 1999; Izumi *et al* (2001); Nakakubo *et al*, 2002). However, the expression of EBAG9 and its clinical significance have not been examined in epithelial ovarian cancer. Therefore, in this study, we examined the expression of EBAG9 in human epithelial ovarian cancer using immunohistochemistry, reverse transcription–polymerase chain reaction (RT–PCR) and immunoblotting. We also evaluated the correlations of EBAG9 immunoreactivity with various clinicopathological parameters and ER status.

## MATERIALS AND METHODS

We studied a total of 90 cases of common epithelial ovarian carcinoma. Information regarding age, performance status on admission, histology, stage, grade, residual tumour after primary surgery and overall survival was retrieved from the review of patient charts. The median follow-up time of the patients in this study was 54 months (18–112 months). Of 90 patients, 76 (84.4%) received platinum-containing chemotherapy after operation. Performance status was defined according to WHO criteria (World Health Organization, 1979). Histology, stage and grade were determined according to FIGO (International Federation of Gynecology and Obstetrics) criteria (Shimizu *et al*, 1998). Residual disease was determined by the amount of unresectable tumour left following primary cytoreductive surgery. Optimal cytoreduction was defined as no gross residual tumour greater than 2 cm in diameter, whereas suboptimal cytoreduction was defined as any gross residual disease remaining greater than 2 cm in diameter. Overall survival was calculated from the time of initial surgery to death, or the date of last contact. Survival times of patients still alive or lost to follow-up were censored in December 2002. All of these archival specimens were retrieved from the surgical pathology files at Tohoku University Hospital, Sendai, Japan. These specimens were all fixed in 10% formalin and embedded in paraffin. Among these 90 cases, 22 cases were available for examination by RT–PCR analysis. These specimens were dissected immediately into small pieces following gross dissection, quickly transferred to liquid nitrogen, and then stored at −80°C until further use. The research protocol was approved by the ethics committee of Tohoku University Graduate School of Medicine, Sendai, Japan.

OVCAR3, Caov3, SKOV3, TOV112D, TOV21G, OV90 and ES2 (adenocarcinoma: OVCAR3, SKOV3; serous adenocarcinoma: Caov3, OV90; clear cell adenocarcinoma: TOV21G, ES2; endometrioid adenocarcinoma: TOV112D) cell lines were purchased from American Type Culture Collection. JHOS2, JHOS3, HTOA, OMC3 and JHOC5 (serous adenocarcinoma: JHOS2, JHOS3, HTOA; mucinous adenocarcinoma: OMC3; clear cell adenocarcinoma: JHOC5) cell lines were purchased from Riken cell bank (Tsukuba, Japan). Cell lines were maintained in DMEM/F12 (Invitrogen, CA), supplemented with 10% foetal bovine serum and 1% penicillin/streptomycin (Invitrogen, CA), and incubated in 5% CO_2_ at 37°C.

### Immunohistochemistry

Immunohistochemical analysis was performed using the streptavidin–biotin amplification method using a Histofine Kit (Nichirei, Tokyo, Japan), and have been previously described in detail (Akahira JI *et al*, 2001, Akahira J *et al*, 2001). Oestrogen receptor-binding fragment associated gene 9 antibody was a rabbit polyclonal antibody against a GST-EBAG9 fusion protein (Tsuchiya *et al*, 2001). The characterisation of this antibody was confirmed by Western blotting (Suzuki *et al*, 2001). Monoclonal antibody for ER*α* was purchased from Immunotech (Marseille, France). For antigen retrieval, the slides were heated in an autoclave at 120°C for 5 min in citric acid buffer (2 mM citric acid and 9 mM trisodium citrate dyhydrate, pH 6.0). The dilutions of primary antibodies for EBAG9 and ER*α* were 1 : 200 and 1 : 2, respectively. The antigen–antibody complex was visualised with 3,3′-diaminobenzidine (DAB) solution (1 mm DAB, 50 mM Tris-HCl buffer (pH 7.6) and 0.006% H_2_O_2_), and counterstained with haematoxylin. The ER-positive breast carcinoma tissue was used as a positive control for EBAG9 (Suzuki *et al*, 2001). As negative controls, 0.01 M phosphate-buffered saline (PBS) and normal mouse IgG were used in place of primary antibodies. No specific immunoreactivity was detected in these tissue sections.

### Scoring of immunostaining

For statistical analyses of EBAG9 immunoreactivity, carcinomas were classified independently by two of the authors (MA and JA) into two groups: +, positive carcinoma cells; and −, no immunoreactivity. Cases with disconcordant results among the observers were re-evaluated. For evaluation of ER*α* immunoreactivity, labeling index (LI) was obtained in carcinoma cells as described by Sasano *et al* (1996). In brief, two of the authors (JA and TM) independently evaluated at least 500 carcinoma cells microsopically and the percentage of immunoreactivity was determined. In the present study, interobserver differences were less than 5%, and the mean of the two values was obtained.

### Reverse transcription–PCR

Total RNA was isolated from tissues by phenol–chloroform extraction using Isogen (Nippon Gene, Japan), and was treated by DNase I (Roche, Germany). The RT–PCR kit (SUPERSCRIPT Preamplification system, Invitrogen) was employed and cDNA synthesis was carried out according to the instructions. cDNAs were synthesised from 5 *μ*g of total RNA using random hexamer and RT was carried out for 50 min at 42°C with SUPERSCRIPT II reverse transcriptase. After an initial 1-min denaturation step at 94°C, 35-cycle PCRs were carried out on a DNA thermal cycler (PTC-200 DNA Engine, MJ Research, Inc., USA) under the following conditions: 1-min denaturation at 94°C, 1-min annealing at 58°C for EBAG9, 62°C for ER*α* and 2-min extension at 72°C. Primers for PCR reactions were as follows: EBAG9: 5′sense – GCTACACAAGATTCTGCCT and 3′ antisense – CTTCTTCATTAGCCGTTGTG (680–892, 213 bp); ER*α*: 5′ sense – AAGAGCTGCCAGGCCTGCC and 3′ antisense – TTGGCAGCTCTCATGTCTCC (702–869, 168 bp); *β*-actin: 5′ sense – CCAACCGCGAGAAGATGAC and 3′ antisense – GGAAGGAAGGCTGGAAGAGT (382–841, 459 bp). In initial expreriments, following amplification, PCR products were purified and subjected to direct sequencing to verify amplification of the correct sequences (ABI prism 310 Genetic Analyzer, Applied Biosystems, CA, USA). *β*-Actin primers were utilised as positive controls. Negative controls without RNA and without reverse transcriptase were also performed.

### Immunoblotting

Cells were grown to 70% confluence in 10-cm plates and after removal of culture medium with PBS, whole-cell protein was extracted by conventional method. The protein concentration was measured by Model 680 microplate reader (Biorad, USA) using BradFord reagent (Biorad). In all, 20 *μ*g of protein of each sample was mixed with an equal volume of 2 × concentrated SDS–polyacrylamide gel electrophoresis (SDS–PAGE) sample buffer, boiled, and then electrophoresed on 7% ready-made gels containing SDS (Mini Protian II Western blotting system, Biorad). Proteins were then transferred to nitrocellulose membrane (Hybond PDVF, Biorad). The membranes were incubated in blocking solution (PBS containing 5% nonfat milk and 0.05% Tween-20), and then incubated in 1 : 200 dilution of EBAG9 antibody (1 : 2 for ER*α* and 1 : 1000 for Actin) in blocking solution overnight at 4°C. After incubation with HRP-rabelled anti-rabbit IgG (Vector Laboratories, Inc., USA), the antigen–antibody complex was visualised with ECL system (Amersham, Germany). MCF-7 breast cancer cell line was used as a positive control (Watanabe *et al*, 1998). Actin (Ab-1, Oncogene) was used as internal positive controls.

### Statistical analysis

Statistical analysis was performed using Stat View 5.0 (SAS Institute Inc., NC, USA) software. The statistical significance between EBAG9 and characteristics of the patients was evaluated using Mann–Whitney *U*-test, Kruskal–Wallis and Scheffe analysis. The correlation between EBAG9 and ER*α* immunoreactivity was also assessed using Mann–Whitney *U*-test. Univariate analysis of prognostic significance for prognostic factors was performed using a log-rank test, after each survival curve was obtained by the Kaplan–Meier method. All patients who could be assessed were included in the intention-to-treat analysis. A result was considered significant when the *P*-value was less than 0.05.

## RESULTS

Results of immunohistochemistry and their correlation with clinicopathological parameters are summarised in Table 1

**Table 1:** Association between EBAG9 immunoreactivity and clinicopathological parameters in human ovarian cancer

	**EBAG9 immunoreactivity**		
	**+(*n*=46)**	**–(*n*=44)**	***P*-value**
Age (years)	52.8±1.8	49.5±1.5	NS
			
*Histological type*			
Serous	27	14	
Mucinous	7	9	
Endometrioid	3	10	
Clear cell	9	11	0.0402
			
*Histological grade*			
Grade 1	15	16	
Grade 2	11	15	
Grade 3	12	6	NS
			
*Stage*			
I,II	17	27	
III,IV	29	17	0.0206
			
*Residual tumour*			
<2 cm	27	34	
>2 cm	19	10	NS
			
*PS*			
0,1	30	32	
2,3,4	16	12	NS
			
ER LI	18.8	11.4	<0.0001

Performance status score: 0=asymptomatic and fully active; 1=symptomatic, fully ambulatory, restricted in physically strenous activity; 2=symptomatic, ambulatory, capable of self-care, more than 50% of waking hours are spent out of bed; 3=symptomatic, limited self-care, spends more than 50% of time in bed, but not bedridden; 4=completely disabled, no self-care, bedridden.

**Figure 1:**
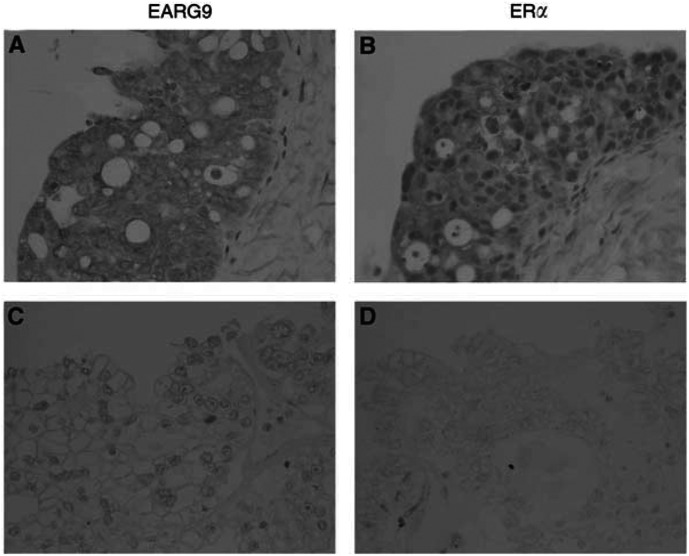
Serial sections of positive and negative cases of immunohistochemistry for EBAG9 and ER*α* in epithelial ovarian carcinoma (**A**,**B**) a case of serous adenocarcinoma, positive for both EBAG9 and ER*α*; (**C**,**D**) a case of clear-cell adenocarcinoma, negative for both EBAG9 and ER*α*). Immunoreactivity for EBAG9 was detected on the surface, and in the cytoplasm of epithelial ovarian cancer tissues. ER-*α* immunoreactivity was confined exclusively to the nuclei of tumour cells.

. Immunoreactivity for EBAG9 was detected on the surface and in the cytoplasm of epithelial ovarian cancer tissues. Oestrogen receptor *α* immunoreactivity was confined exclusively to the nuclei of tumour cells (Figure 1

). The number of cases immunopositive for EBAG9 was 46 out of 90 cases (51.1 %). The median LI for ER*α* was 12.8 % (0–85.2%). As shown in Table 1, EBAG9 expression was significantly higher in serous histology (*P*=0.0402) and advanced disease (*P*=0.0206). There was no significant relationship between EBAG9 immunoreactivity and patient age, histological grade, residual tumour or performance status. There was a highly significant correlation between EBAG9 immunoreactivity and ER*α* LI (*P*<0.0001).

Results of univariate analysis of prognostic significance for each variable, with respect to survival, are summarised in Table 2

**Table 2:** Univariate analysis of overall survival

**Variable**	***P*-value**
EBAG9 immunoreactivity (+ *vs* −)	0.689
Histological type	0.0177
Histological grade	0.0085
Stage	<0.0001
Residual tumour	<0.0001

. Among the clinicopathological factors examined, those significantly associated with overall survival were histology, grade, stage and residual tumour. No significant relationship was detected between EBAG9 immunoreactivity and overall survival (*P*=0.689).

Relationships between EBAG9 immunoreactivity and EBAG9, ER*α* mRNA in 22 cases are summarised in Table 3

**Table 3:** Association between EBAG9 immunoreactivity and RT–PCR

		**EBAG9 immunoreactivity**	
		+	**–**
EBAG9 mRNA	+	16	4
	−	0	2
			
ER*α* mRNA	+	9	2
	−	0	6

**Figure 2:**
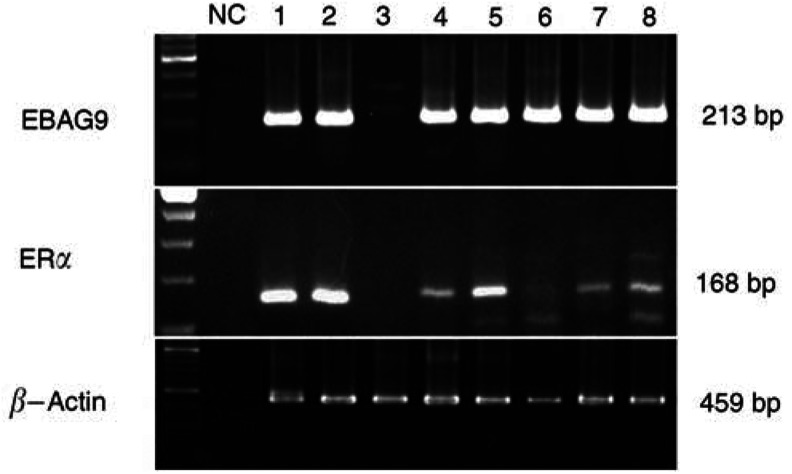
Representative results of RT–PCR for total RNA extracted from epithelial ovarian carcinoma tissues. Bands of the correct size for EBAG9 (213 bp), ER*α* (168 bp) and *β*-actin (459 bp) were detected in each histological subtype of ovarian cancer (1,2: serous; 3,4: mucinous; 5,6: endometrioid; 7,8: clear cell). *β*-Actin was used as a positive control and NC as a negative control. Note that all of the cases that were positive for ER*α* mRNA were also positive for EBAG9 mRNA.

. Oestrogen receptor-binding fragment associated gene 9 mRNA was positive in 20 cases. In four cases, EBAG9 immunoreactivity was not detected although its mRNA was present. In all of the cases that were positive for ER*α* mRNA (*n*=11), they were also positive for EBAG9 mRNA (Figure 2

).

**Figure 3:**
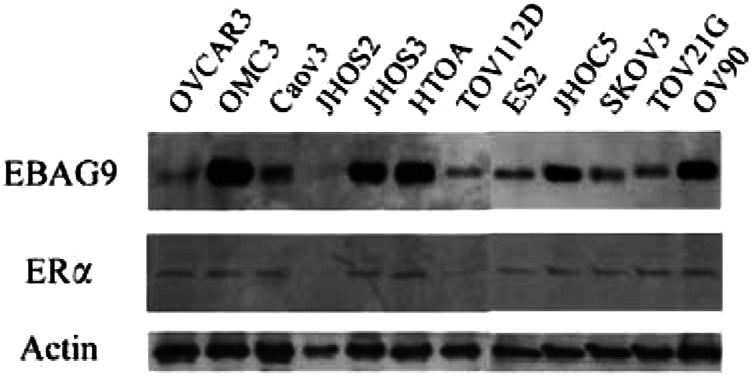
Results of immunoblotting of ovarian cancer cell lines are shown. Actin was used as internal positive controls.

The result of immunoblotting is shown in Figure 3

. Immunoreactive bands corresponding to EBAG9 and ER*α* were detected in 11 out of 12 and 10 out of 12 of ovarian cancer cell lines, respectively. JHOS2, which was negative for EBAG9, was also negative for ER*α*.

## DISCUSSION

In the present study, EBAG9 immunoreactivity was detected in 46 out of 90 epithelial ovarian carcinomas (51.1%). The expression of EBAG9 was significantly associated with advanced disease, although it turned out not to be correlated with prognosis.

It is well recognised that human cancer tissues are infiltrated with tumour-infiltrating lymphocytes (TIL) (Balch *et al*, 1990), a phenomenon known to be a manifestation of the host immune reaction to cancer cells (Rosenberg, 1996). Tumour-infiltrating lymphocytes has been reported to be associated with improved prognosis of some carcinomas, including lung (DiPaola *et al*, 1977) and colon (Naito *et al*, 1998) carcinomas. In ovarian tumour, the degree of lymphocyte infiltration was reported to be associated with the patients' survival rate, clinical stage, grade and histological type (Ma and Gu, 1991). Nakashima *et al* (1999) recently reported that activated CD3+ T lymphocytes express a putative receptor for EBAG9/RCAS1. This receptor expression was enhanced by activation of the lymphocytes, and when these receptor-positive cells were cultured with EBAG9/RCAS1 peptides, their growth was strongly suppressed, and they were eventually led to cell death by apoptosis (Nakashima *et al*, 1999). In breast carcinoma, EBAG9 immunoreactivity was inversely associated with the degree of intratumoral infiltration of mononuclear cells or CD3+ T lymphocytes (Suzuki *et al*, 2001). These results suggested that tumour cells might have evaded immune surveillance by expressing EBAG9/RCAS1, which suppressed clonal expansion and induced apoptosis of receptor-positive immune cells. Although the precise mechanism of immune evasion remains uncertain, the expression of EBAG9/RCAS1 may be a factor related to the escape mechanism of cancer cells from the host immune system.

Several authors reported that expression of RCAS1 was associated with poor prognosis and/or advanced disease in human cancer. Izumi *et al* (2001) reported that RCAS1 expression was positive for 48 of 102 non-small-cell lung carcinoma patients (47.1%) and was significantly correlated with advanced stage, poor differentiation and poor prognosis. Ito *et al* (2003) reported that RCAS1 overexpression was more frequently observed in anaplastic carcinomas than well-differentiated carcinoma in thyroid cancer. In pancreatic ductal adenocarcinoma, RCAS1 expression was demonstrated in 77 of 80 cases (96%) and was an independent prognostic factor (Hiraoka *et al*, 2002). In gynaecological malignancies, Kaku *et al* (1999) reported that patients with high RCAS1 expression showed significantly worse overall survival than those with low expression in adenocarcinoma of uterine cervix. In addition, Sonoda *et al* (1998) reported that RCAS1 was not detected in the normal uterine cervix or ovarian tissue, but strongly expressed in uterine endometrial adenocarcinomas, ovarian adenocarcinomas (Sonoda *et al*, 1996; Sonoda *et al*, 2000) and cervical squamous cell carcinomas. Recently, Suzuki *et al* (2001) reported that EBAG9 immunoreactivity was detected in 82 of 91 in breast carcinoma (90.1%), although it was not associated with clinicopathological parameters. Others reported that EBAG9 gene was consistently expressed in breast cancer cell line, and might play a specific role in early stages of breast carcinogenesis (Tsuneizumi *et al*, 2001). To our knowledge, this is the first report that evaluated the relationships between EBAG9/RCAS1 and clinicopathological parameters in epithelial ovarian cancer. Our results, together with previous reports, suggest that ovarian cancer that expresses EBAG9 may have invasive and progressive characteristics.

There was a strong correlation between EBAG9 and ER*α* immunoreactivity in ovarian cancer tissues (*P*<0.0001) and cell lines in this study. Also, EBAG9 immunoreactivity was associated with serous histology. Moreover, all of the cases that were positive for ER*α* mRNA, were also positive for EBAG9 mRNA, suggesting that the regulation of EBAG9 may be under oestrogen control in ovarian epithelial carcinoma. Oestrogen receptor-binding fragment associated gene 9 was isolated utilising a genomic-binding site cloning method from a cDNA library of MCF-7 human breast cancer cell (Watanabe *et al*, 1998), which expresses ER*α* and low level of ER*β* (Vladusic *et al*, 2000). Transfection analyses have demonstrated that the nucleotide sequences between −86 and −36 contains an ERE in the 5′-promoter region of the EBAG9 gene (Ikeda *et al*, 2000). mRNA levels of EBAG9 in MCF-7 cells are significantly increased within 6 h of oestrogen treatment, an effect that is mediated by the binding of ER*α* to the ERE in the promoter of the EBAG9 gene (Ikeda *et al*, 2000). On the other hand, Quinn *et al* (1982) reported that serous tumours were more frequently ER-positive than other types of cancers. Results from our present study are consistent with these previous reports, and suggest that EBAG9 is widely distributed in carcinoma cells of human epithelial ovarian carcinoma tissues and cells, maybe especially in serous histology, as a result of oestrogen actions through ER.

In conclusion, the wide distribution of EBAG9 and its relation to advanced disease suggest that this protein may play important roles in epithelial ovarian cancer. Further investigations are required to clarify the precise functions of EBAG9 in epithelial ovarian cancer.

## References

[bib1] Akahira J, Suzuki T, Ito K, Darnel AD, Moriya T, Sato S, Yaegashi N, Okamura K, Sasano H (2001) Expression of 5*α*-reductases in human epithelial ovarian cancer: its correlation with androgen receptor status. Jpn J Cancer Res 92: 926–932 11572759 10.1111/j.1349-7006.2001.tb01182.x PMC5926843

[bib2] Akahira J, Suzuki T, Ito K, Kaneko C, Darnel AD, Moriya T, Okamura K, Yaegashi N, Sasano H (2002) Differential expression of progesterone receptor isoforms A and B in the normal ovary, and in benign, borderline, and malignant ovarian tumors. Jpn J Cancer Res 93: 807–815 12149147 10.1111/j.1349-7006.2002.tb01323.x PMC5927076

[bib3] Akahira JI, Yoshikawa H, Shimizu Y, Tsunematsu R, Hirakawa T, Kuramoto H, Shiromizu K, Kuzuya K, Kamura T, Kikuchi Y, Kodama S, Yamamoto K, Sato S (2001) Prognostic factors of stage IV epithelial ovarian cancer: a multicenter retrospective study. Gynecol Oncol 81: 398–403 11371128 10.1006/gyno.2001.6172

[bib4] Balch CM, Riley LB, Bae YJ, Salmeron MA, Platsoucas CD, von Eschenbach A, Itoh K (1990) Patterns of human tumor-infiltrating lymphocytes in 120 human cancers. Arch Surg 125: 200–205 1689143 10.1001/archsurg.1990.01410140078012

[bib5] Benraad THJ, Friberg CG, Koenders AJM, Kullander S (1980) Do estrogen and progesterone receptors (ER and PR) in metastasizing endometrial cancer predict response to estrogen therapy. Acta Obstet Gynecol Scand 59: 155–159 7405552 10.3109/00016348009154633

[bib6] Bizzi A, Codegoni AM, Landoni F (1988) Steroid receptors in epithelial ovarian cancer: relation to clinical parameters and survival. Cancer Res 48: 6222–6226 3167868

[bib7] DiPaola M, Bertolotti A, Colizza S, Coli M (1977) Histology of bronchial carcinoma and regional lymph nodes as putative immune response of the host to the tumor. J Thorac Cardiovasc Surg 73: 531–537 839842

[bib8] Ehrlich CE, Young PLM, Cleary RE (1981) Cytoplasmic progesterone and estradiol receptors in normal, hyperplastic and carcinomatous endometria: therapeutic implications. Am J Obstet Gynecol 141: 539–546 6457531 10.1016/s0002-9378(15)33275-0

[bib9] Hempling RE, Piver MS, Eltabbakh GH, Recio FO (1998) Progesterone receptor status is a significant prognostic variable of progression-free survival in advanced epithelial ovarian cancer. Am J Clin Oncol 21: 447–451 9781597 10.1097/00000421-199810000-00005

[bib10] Hiraoka K, Hida Y, Miyamoto M, Oshikiri T, Suzuoki M, Nakakubo Y, Shinohara T, Itoh T, Shichinohe T, Kondo S, Kasahara N, Katoh H (2002) High expression of tumor-associated antigen RCAS1 in pancreatic ductal adenocarcinoma is an unfavorable prognostic marker. Int J Cancer 99: 418–423 11992411 10.1002/ijc.10381

[bib11] Ikeda K, Sato M, Tsutsumi O, Tsuchiya F, Tsuneizumi M, Emi M, Imoto I, Inazawa J, Muramatsu M, Inoue S (2000) Promoter analysis and chromosomal mapping of human EBAG9 gene. Biochem Biophys Res Commun 273: 654–660 10873660 10.1006/bbrc.2000.2920

[bib12] Ito Y, Yoshida H, Nakano K, Kobayashi K, Yokozawa T, Hirai K, Matsuzuka F, Matsuura N, Kuma K, Miyauchi A (2003) Overexpression of human tumor-associated antigen, RCAS1, is significantly linked to dedifferentiation of thyroid carcinoma. Oncology 64: 83–89 12457035 10.1159/000066517

[bib13] Izumi M, Nakanishi Y, Yoshino I, Nakashima M, Watanabe T, Hara N (2001) Expression of tumor-associated antigen RCAS1 correlates significantly with poor prognosis in nonsmall cell lung carcinoma. Cancer 92: 446–451 11466701 10.1002/1097-0142(20010715)92:2<446::aid-cncr1341>3.0.co;2-3

[bib14] Kaku T, Sonoda K, Kamura T, Hirakawa T, Sasaki K, Amada S, Ogawa S, Kobayashi H, Nakashima M, Watanabe T, Nakano H (1999) The prognostic significance of tumor-associated antigen 22-1-1 expression in adenocarcinoma of the uterine cervix. Clin Cancer Res 5: 1449–1453 10389931

[bib15] Kaupilla A (1984) Progestin therapy of endometrial, breast and ovarian carcinoma: a review of clinical observations. Acta Obstet Gynecol Scand 63: 441–450 6238499 10.3109/00016348409156700

[bib16] Ma D, Gu MJ (1991) Immune effect of tumor-infiltrating lymphocytes and its relation to the survival rate of patients with ovarian malignancies. J Tongji Med Univ 11: 235–239 1668016 10.1007/BF02888158

[bib17] Masood S, Heitmann J, Nuss RC, Benrubi GI (1989) Clinical correlation of hormone receptor status in epithelial ovarian cancer. Gynecol Oncol 34: 57–60 2737527 10.1016/0090-8258(89)90107-8

[bib18] McGuire WL (1978) Steroid receptors in human breast cancer. Cancer Res 38: 4289–4291 698967

[bib19] Naito Y, Saito K, Shiiba K, Ohuchi A, Saigenji K, Nagura H, Ohtani H (1998) CD8+ T cells infiltrated within cancer cell nests as a prognostic factor in human colorectal cancer. Cancer Res 58: 3491–3494 9721846

[bib20] Nakakubo Y, Hida Y, Miyamoto M, Hashida H, Oshikiri T, Kato K, Suzuoki M, Hiraoka K, Ito T, Morikawa T, Okushiba S, Kondo S, Katoh H (2002) The prognostic significance of RCAS1 expression in squamous cell carcinoma of the oesophagus. Cancer Lett 177: 101–105 11809537 10.1016/s0304-3835(01)00773-x

[bib21] Nakashima M, Sonoda K, Watanabe T (1999) Inhibition of cell growth and induction of apoptotic cell death by the human tumor-associated antigen RCAS1. Nat Med 5: 938–942 10426319 10.1038/11383

[bib36] Quinn MA, Pearce P, Rome R, Funder JW, Fortune D, Pepperell RJ (1982) Cytoplasmic steroid receptors in ovarian tumours. Br J Obstet Gynaecol 89: 754–759 7115640 10.1111/j.1471-0528.1982.tb05104.x

[bib22] Rao BR, Slotman BJ (1991) Endocrine factors in common epithelial ovarian cancer. Endocr Rev 12: 14–26 1851084 10.1210/edrv-12-1-14

[bib23] Rosenberg SA (1996) The immunotherapy of solid cancers based on cloning the genes encoding tumor-rejection antigens. Ann Rev Med 47: 481–491 8712798 10.1146/annurev.med.47.1.481

[bib24] Sasano H, Frost AR, Saitoh R, Harada N, Poutanen M, Vihko R, Bulin SE, Silverberg SG, Nagura H (1996) Aromatase and 17b-hydroxysteroid dehydrogenase type 1 in human breast carcinoma. J Clin Endoclinol Metab 81: 4042–4046 10.1210/jcem.81.11.8923858 8923858

[bib25] Sevelda P, Denison U, Schemper M, Spona J, Vavra N, Salzer H (1990) Oestrogen and progesterone receptor contents as a prognostic factor in advanced epithelial ovarian carcinoma. Br J Obstet Gynecol 97: 706–712 10.1111/j.1471-0528.1990.tb16243.x 2400748

[bib26] Shimizu Y, Kamoi S, Amada S, Hasumi K, Akiyama F, Silverberg SG. (1998) Toward the development of a universal grading system for ovarian epithelial carcinoma. I. Prognostic significance of histopathologic features-problems involved in the architectural grading system. Gynecol Oncol 70: 2–12 9698465 10.1006/gyno.1998.5051

[bib27] Sonoda K, Kaku T, Hirakawa T, Kobayashi H, Amada S, Sakai K, Nakashima M, Watanabe T, Nakano H (2000) The clinical significance of tumor-associated antigen RCAS1 expression in the normal, hyperplastic, and malignant uterine endometrium. Gynecol Oncol 79: 424–429 11104614 10.1006/gyno.2000.5981

[bib28] Sonoda K, Kaku T, Kamura T, Nakashima M, Watanabe T, Nakano H (1998) Tumor-associated antigen 22-1-1 expression in the uterine cervical squamous neoplasias. Clin Cancer Res 4: 1517–1520 9626471

[bib29] Sonoda K, Nakashima M, Kaku T, Kamura T, Nakano H, Watanabe T (1996) A novel tumor-associated antigen expressed in human uterine and ovarian carcinomas. Cancer 77: 1501–1509 8608535 10.1002/(SICI)1097-0142(19960415)77:8<1501::AID-CNCR12>3.0.CO;2-3

[bib30] Suzuki T, Inoue S, Kawabata W, Akahira J, Moriya T, Tsuchiya F, Ogawa S, Muramatsu M, Sasano H (2001) EBAG9/RCAS1 in human breast carcinoma: a possible factor in endocrine-immune interactions. Br J Cancer 85: 1731–1737 11742495 10.1054/bjoc.2001.2176 PMC2363964

[bib31] Tsuchiya F, Ikeda K, Tsutsumi O, Hiroi H, Momoeda M, Taketani Y, Muramatsu M, Inoue S (2001) Molecular cloning and characterization of mouse EBAG9, homolog of a human cancer associated surface antigen: expression and regulation by estrogen. Biochem Biophys Res Commun 1: 2–10 10.1006/bbrc.2001.4892 11374862

[bib32] Tsuneizumi M, Emi M, Nagai H, Harada H, Sakamoto G, Kasumi F, Inoue S, Kazui T, Nakamura Y (2001) Overrepresentation of the EBAG9 gene at 8q23 associated with early-stage breast cancers. Clin Cancer Res 7: 3526–3532 11705872

[bib33] Vladusic EA, Hornby AE, Guerra-Vladusic FK, Lakins J, LupuR (2000) Expression and regulation of estrogen receptor beta in human breast tumors and cell lines. Oncol Rep 7: 157–167 10601611 10.3892/or.7.1.157

[bib34] Watanabe T, Inoue S, Hiroi H, Orimo A, Kawashima H, Muramatsu M (1998) Isolation of estrogen-responsive genes with a CpG island library. Mol Cell Biol 18: 442–449 9418891 10.1128/mcb.18.1.442 PMC121513

[bib35] World Health Organization (1979) Handbook for Reporting Results of Cancer Treatment, WHO publication no. 48. Geneva, Switzerland: World Health Organization

